# Next‐generation sequencing reveals unique combination of mutations in cis of *CSF3R* in atypical chronic myeloid leukemia

**DOI:** 10.1002/jcla.23064

**Published:** 2019-11-06

**Authors:** Jae Won Yun, Jung Yoon, Chul Won Jung, Ki‐O Lee, Jong Won Kim, Sun‐Hee Kim, Hee‐Jin Kim

**Affiliations:** ^1^ Department of Laboratory Medicine and Genetics Samsung Medical Center Sungkyunkwan University School of Medicine Seoul Korea; ^2^ Department of Internal Medicine Samsung Medical Center Sungkyunkwan University School of Medicine Seoul Korea; ^3^ Samsung Medical Center Samsung Biomedical Research Institute Seoul Korea

**Keywords:** atypical chronic myeloid leukemia, CSF3R, next generation sequencing, SETBP1, U2AF1

## Abstract

**Background:**

Atypical chronic myeloid leukemia (aCML) is a hematologic disorder characterized by leukocytosis with increased dysplastic neutrophils and their precursors. In CSF3R gene, the activation mutation including T618I is frequently reported in aCML but is rarely accompanied by truncation mutations. Herein, we report a unique aCML patient with two CSF3R mutations (T618I and Y779*) in the same DNA strand.

**Methods:**

High‐coverage next‐generation sequencing for 40 genes related with myeloid leukemia was performed. Sanger sequencing was performed to confirm CSF3R mutations. To confirm whether two CSF3R mutations are in cis or not, TA cloning was used. Clinical information and bone marrow pathology were reviewed by two hematopathologists.

**Results:**

In the patient diagnosed with aCML in bone marrow study, two *CSF3R* mutations, (T618I and Y779*) a *SETBP1* mutation (G870S) and an *U2AF1* mutation (Q157P), were identified by high‐coverage next‐generation sequencing. The two CSF3R mutations were confirmed to be located in the same DNA strand by TA cloning, indicating that the two mutations are harbored in one malignant clone. The *SETBP1* mutation is known to be related with poor prognosis in aCML. Likewise, the patient was refractory to hydroxyurea and showed disease progression. Additionally, we discussed the potential therapeutic targets by reviewing the molecular profile of the patient.

**Conclusion:**

We believe that the accurate diagnosis and maximum therapeutic chance could be achieved by profiling the mutations and their characteristics.

## 1 INTRODUCTION

Atypical chronic myeloid leukemia (aCML) is a rare disease belonging to the myelodysplastic/myeloproliferative neoplasms (MDS/MPN) group according to the 2016 World Health Organization (WHO) classification of hematologic malignancies. While aCML shows sharing hematologic findings with CML with *BCR*‐*ABL1*, the major differential diagnostic points include the absence of *BCR*‐*ABL1* rearrangement and presence of prominent dysgranulopoiesis. Clinically, aCML is an aggressive disease with a poor prognosis.^1^ For these reasons, there is an unmet need for discovering molecular markers to expand treatment options and to monitor the disease. Recently, deep sequencing using next‐generation sequencing (NGS) is applied in a variety of diseases and can help solve the unmet need. Using NGS, multiple cancer genes are tested with a high mutation detection sensitivity. Driver and key mutations found by NGS could be useful therapeutic targets.^2^, ^3^

In aCML, poor prognosis is known to be related with female gender, older age (>65 years), leukocytosis >50 × 10^9^/L, and the presence of circulating precursors.^4^ In molecular level, although mutations from several genes including *KRAS, NRAS, SETBP1, CSF3R*, *ASXL1,* and *ETNK1* are frequently observed in aCML,^5^ mutations from *SETBP1* and *ASXL1* are considered to be associated with poor prognosis till now.^5^, ^6^, ^7^ In therapeutic aspect, mutations in genes related with JAK‐STAT, MAPK, ROCK, and SRC family‐TNK2 kinase signaling are gaining attention as therapeutic targets recently.^5^, ^8^, ^9^

Herein, we describe a case of aCML with a unique molecular profile revealed by NGS. The patient had double mutations of Y779* (first case in atypical CML) and T618I (known mutation) in the *CSF3R* gene. By using TA cloning, we also found that the two mutations are located in cis, suggesting that the two mutations are on the same RNA transcript of one malignant clone. In addition, two gain‐of‐function mutations in the *SETBP1* and *U2AF1* genes were found with high variant allele frequencies (VAFs). We discussed the biological, diagnostic, and therapeutic significance of these mutations.

## 2 MATERIALS AND RESULTS

### 2.1 Patient

**Figure 1:**
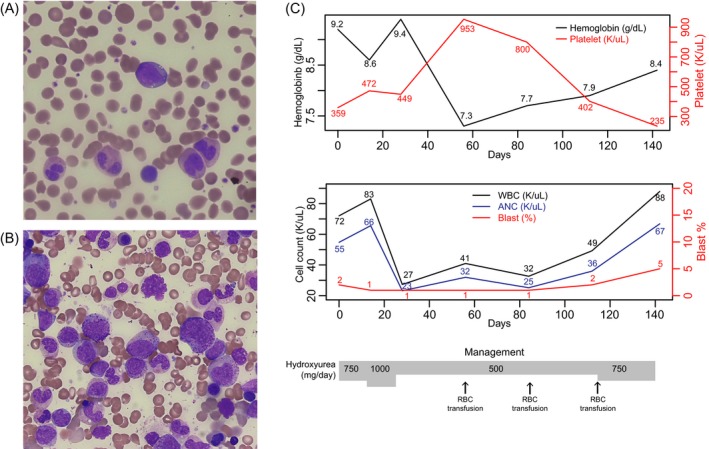
Peripheral blood smear (A) and bone marrow aspirate (B) in Wright and Giemsa stains. A blast and dysplastic granulocytes were shown (A) and granulocytic proliferation and granulocytic dysplasia in bone marrow (B). C, Laboratory findings including hemoglobin, platelet, white blood cell count, and blast count during the 140 days of hydroxyurea therapy

The patient was a 75‐year‐old woman transferred from an outside hospital because of abnormal CBC with leukocytosis. She experienced anorexia, sweating, and weight loss. Physical examination revealed mild splenomegaly with right upper quadrant abdominal discomfort. CBC showed Hb 9.2 g/dL, white blood cells (WBC) 72 K/µL with blasts 2% and granulocytic precursors 29%, and platelets 359 K/µL (Figure 1). Peripheral blood smear demonstrated dysplastic features in the granulocytic series (Figure 1). Bone marrow (BM) study was performed and revealed granulocytic proliferation with dysplasia (Figure 1), increased megakaryocytes, and focal fibrosis. Chromosome analysis showed 47,XX,+14[19]/46,XX[1]. Molecular genetic studies were performed to screen *BCR*/*ABL1*, *PDGFRA*, *PDGFRB*, and *FGFR1* rearrangements, and point mutations of *JAK2*, *CALR,* and *MPL*, and the results were all negative. Collectively, the patient was diagnosed as having aCML based on the 2016 WHO diagnostic criteria.^10^ After patient started hydroxyurea therapy, leukocytosis and blast count were decreased initially (Figure 1C). However, leukocytosis and blast count started to be increased after 3 months of hydroxyurea therapy, suggesting the refractoriness to the medication.

### 2.2 Next‐generation sequencing and gene mutations

Next‐generation sequencing was performed to detect gene mutations other than the aforementioned major driver mutations in MPN. DNA was extracted using the Promega DNA Extraction Kit (Promega) following the manufacturer's instructions. Isolation of DNA was performed using the RecoverAll Total Nucleic Acid Isolation Kit (Thermo Fisher Scientific). The library preparation was conducted using the Ion Chef System (Thermo Fisher Scientific, San Francisco, CA, USA) per the manufacturer's instructions. Sequencing was conducted on the Ion S5 XL Sequencer using the Ion 530 Chip and Ion 530 kit‐Chef (Thermo Fisher Scientific). Alignment and base calling were performed using the Ion Reporter (Version 5.6.0) with Oncomine™ Myeloid Research‐530‐w2.3.1 workflow based on the reference genome hg19. Variant detection and annotation were performed using Ion Reporter software (Version 5.6). The DNA panel from Oncomine^TM^ myeloid research assay was designed to test the following genes: *ABL1*, *ASXL1*, *BCOR*, *BRAF*, *CALR*, *CBL*, *CEBPA*, *CSF3R*, *DNMT3A*, *ETV6*, *EZH2*, *FLT3*, *GATA*2, *HRAS*, *IDH1*, *IDH2*, *IKZF1*, *JAK2*, *KIT*, *KRAS*, *MPL*, *MYD88*, *NF1*, *NPM1*, *NRAS*, *PHF6*, *PRPF8*, *PTPN11*, *RB1*, *RUNX1*, *SETBP1*, *SF3B1*, *SH2B3*, *SRSF2*, *STAG2*, *TET2*, *TP53*, *U2AF1L5*, *WT1*, and *ZRSR2*. As a result, we detected two pathogenic variants in the *CSF3R* gene, one variant each in *SETBP1* and in *U2AF1* (Table 1). The two CSF3R mutations were a truncation mutation c.2337T>G (Y779*) in receptor cytoplasmic domain and the well‐known activating mutation c.1853C>T (T618I) affecting the extracellular domain. The mutation in *SETBP1* was c.2608G>A (G870S), and the mutation in *U2AF1* was c.470A>C (Q157P). The variant allele frequencies (VAFs) of *CSF3R* Y779*, *CSF3R* T618I, *SETBP1* G870S, and *U2AF1* Q157P were 48.4%, 45.9%, 48.9%, and 47.2%, respectively (Table 1). All mutations were confirmed by Sanger sequencing.

**Table 1:** Mutations revealed by next‐generation sequencing

Gene	cDNA change	AA change	VAF (%)	Coverage
CSF3R	c.2337T>G	p.Tyr779Ter	48.4	1989
CSF3R	c.1853C>T	p.Thr618Ile	45.9	1998
SETBP1	c.2608G>A	p.Gly870Ser	48.9	1999
U2AF1	c.470A>C	p.Gln157Pro	47.2	1998

Abbreviation: VAF, variant allele frequencie.

### 2.3 TA cloning for *CSF3R* double mutations

**Figure 2:**
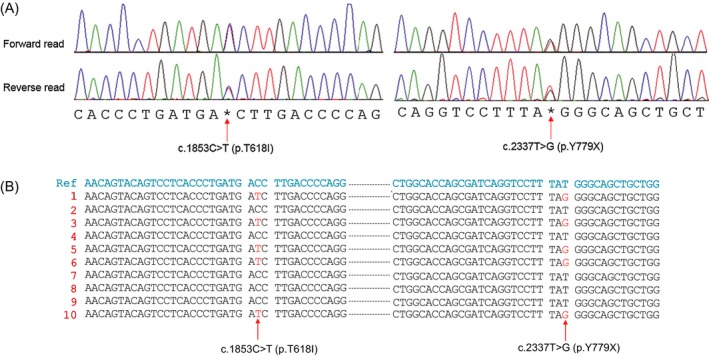
Sanger sequencing confirmation for two CSF3R mutations (A). TA cloning to identify whether the two CSF3R mutations were in the same DNA strand or not (B). In the lanes of 1, 3, 5, 6, and 10, c.1853C>T and c.2337T>G were found simultaneously, while no CSF3R mutations were found in other lanes. These findings suggest that the two CSF3R mutations are located in the same DNA strand, finally indicating that the two mutations are harbored in the same leukemic clone

We additionally performed TA cloning to determine whether the 2 mutations of *CSF3R* were on the same mutant allele. PCR was performed by using SimpliAmp thermal cycler (Life Technologies) (primer sequence and reaction conditions are available upon request), and the PCR products were ligated into a pGEM‐T easy vector system (Promega). Ten subcloned DNA was sequenced (Cosmo Genetech Inc, Seoul, Korea), and the sequence data were analyzed using DNASTAR™ Lasergene software (Thermo Fisher). As a result, the sequences from colonies 1, 3, 5, 6, and 10 harbored both T618I and Y779* mutations, while those from the remaining colonies had no mutations. These results demonstrated that the two mutations were on the same allele (Figure 2B).

## 3 DISCUSSION

In this report, we described a patient with aCML having a unique genetic profile: double mutations in *CSF3R*, T618I, and T779*, along with *SETBP1* G870S, *U2AF1* Q157P, and trisomy 14. Unlike CML with *BCR*‐*ABL1,* aCML has is no molecular hallmark. The detection of multiple gene mutations was possible through NGS covering a panel of recurrently mutated genes in myeloid neoplasms, and we could detect those mutations.

**Figure 3:**
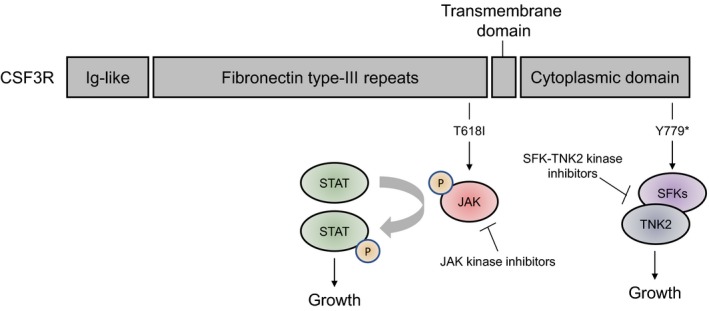
Location of two mutations in CSF3R. The T618I is related with JAK signaling, and the signal could be inhibited by JAK kinase inhibitor. The CSF3R truncation mutation (Y779*) is related with proliferation signaling via SFK‐TNK2, and this proliferation signaling is potentially inhibited by SFK‐TNK2 kinase inhibitor

The CSF3R gene encodes the receptor for colony‐stimulating factor 3, a cytokine that controls the production and differentiation of granulocytes. The pathogenic variant in CSF3R is commonly found in hematologic malignancies including acute myeloid leukemia, chronic neutrophilic leukemia, and atypical CML.^11^ Double mutations in *CSF3R*, one in the domain and the other in the C‐terminal as in our patient, are previously reported in a small proportion of aCML. However, the T779* mutation of *CSF3R* has been reported in congenital neutropenia or acute myeloid leukemia as a sole mutation.^12^, ^13^ Thus, the combination of T618I and Y779* mutations is novel, and our patient with aCML is the first report. Y779* co‐existing with T618I is novel in aCML and seemed to be associated with delayed receptor internalization based on the location of the mutation.^14^ The two mutations were suggested to be on the same allele based on the VAF information, and TA cloning results confirmed the status. First, the malignant clone harbors both mutations simultaneously in the same DNA strand. Hence, this finding helps in establishing the strategy for a molecular target therapy. The T618I is known to be related with cell growth via JAK‐STAT signaling and the JAK‐STAT signaling could be inhibited by JAK inhibitors in this intractable disease (Figure 3). Additionally, the truncation mutation, Y779*, could be related with cell growth signaling via SFK‐TNK2 and this could be inhibited by SFK‐TNK2 kinase inhibitors. Finally, the malignant clone has two potential therapeutic targets. Second, it can be inferred that the two mutations, T618I and Y779*, play a synchronistic or complementary role in forming a tumor clone via activation and delayed receptor internalization of CSF3R.

The *SETBP1* gene encodes SET binding protein 1, which could bind the SET nuclear oncogene which is involved in DNA replication. The gain‐of‐function mutation of *SETBP1* in our patient, G870S, is located in the SKI homologous region and has been reported mostly in chronic myelomonocytic leukemia, myelodysplastic syndrome, and acute myeloid leukemia based on the COSMIC database.^15^ Furthermore, G870S is known to be associated with higher WBC counts and worse prognosis.^6^ Likewise, our patient was refractory to hydroxyurea treatment and showed disease progression with high leukocyte count and increasing blast count (Figure 1C). The *U2AF1* gene encodes a U2 small nuclear RNA auxiliary factor 1, which plays a critical role in RNA splicing. The gain‐of‐function mutation of *U2AF1* detected in our patient was Q157P. The Q157 residue is located in the second zinc finger domain,^16^ and Q157P is a known pathogenic mutation that alters splicing of many important genes in myeloid disorders,^17^ while the prognostic significance has not been fully investigated in aCML. Interestingly, several molecular features in the patient are considered to be related with dysplasia, which is a typical bone marrow finding in aCML *SETBP1* mutation is reported to be related with dysplastic morphology in MDS/MPN.^18^ The mutation in aCML having dysplasia as one of diagnostic hallmark is frequently found with 24%.^6^ The gain‐of‐function mutation in *U2AF1* is thought to contribute dysplastic hematopoiesis. In the report, the authors described that hundreds of exons were differentially spliced with association of *U2AF1* mutation and that the phenomenon could be related with dysplasia and tumorigenesis.^17^ In addition, sole trisomy 14 was found in chromosome analysis. The chromosomal aberration is also reported to be related with dysplastic blood cancer, while further investigation is needed for its biology and clinical significance.^19^

In the aspect of treatment, there was additional chance of a clinical trial with several therapeutic targets, although the patient was lost to follow‐up with disease progression, unfortunately. For T618I of *CSF3R*, ruxolitinib showed efficacy according to several case reports.^9^, ^20^, ^21^ The G870S of *SETBP1* is reported to imply ruxolitinib unresponsiveness.^22^ On the other hand, the *SETBP1* mutation was suggested as a novel target of fingolimod (FTY720) in an in vitro study.^23^

For differential diagnosis of aCML with CSF3R mutation, chronic neutrophilic leukemia (CNL) is an important disease in that *CSF3R* mutations are more frequent in CNL. The *CSF3R* mutation frequencies are reported to be 43% and less than 10% in CNL and aCML, respectively.^10^, ^24^ In the diagnostic criteria, the main difference between the two diseases is that increased neutrophil precursors more than 10% of WBC with dysplasia were observed in aCML while not in CNL.^10^ In molecular aspect, the genetic drivers seemed to be more heterogenous in aCML than CNL.^7^ Atypical CML is associated with the presence of *SETBP1* and/or *ENTK1* mutations while CNL is associated the presence of *CSF3R* mutations.^10^ In an analysis of 14 CNL and 58 aCML patients, Meggendorfer et al reported that *CSF3R* mutations are statistically more frequent in CNL.^24^ In the study, the mutation frequencies of *ASXL1, SETBP1*, *SRSF2,* and *TET2* are not rare in both aCML and CNL, although their frequencies are seemed to be higher in aCML. As in our case, morphological findings such as the proportion of neutrophil precursors and dysplasia are critical in differential diagnosis between CNL and aCML with mutated CSF3R till now. Molecular markers discriminating the two diseases need to be further investigated.

In summary, we detected double mutations of *CSF3R* in cis with gain‐of‐function mutations of *SETBP1* and *U2AF1* in our patient with aCML through deep sequencing and TA cloning. We believe that the accurate diagnosis and maximum therapeutic chance could be achieved by profiling the mutations and their characteristics.

## CONFLICT OF INTEREST

The authors declare that they have no competing interests.

## Data Availability

All the information about the case report is available from the corresponding author on reasonable request.
